# Psychometric validation of the Chinese versions of EQ-5D-Y-3L and the experimental EQ-TIPS in children and adolescents with COVID-19

**DOI:** 10.1007/s10198-024-01710-1

**Published:** 2024-07-27

**Authors:** Wenjing Zhou, Yaqin Li, Jan Busschbach, Michael Herdman, Zhihao Yang, Yanming Lu

**Affiliations:** 1 Department of Paediatrics, Renji Hospital, School of Medicine, Shanghai Jiaotong University, No. 2000, Jiangyue Road, Shanghai, China; 2 Department of Psychiatry, Section Medical Psychology and Psychotherapy, Erasmus Medical Center, Rotterdam, The Netherlands; 3 Saw Swee Hock School of Public Health, National University of Singapore, Singapore, Singapore; 4 Health Services Management Department, Guizhou Medical University, Guiyang, China

**Keywords:** COVID-19, EQ-5D-Y-3L, EQ-TIPS, Health-related quality of life, Validation, I19

## Abstract

**Objectives:**

Respiratory infectious diseases like COVID-19 profoundly impacts the health of children and adolescents, but validated instruments to measure their impacts on health-related quality of life (HRQoL) are lacking. The EQ-5D-Y-3L, widely used for youth HRQoL, now features a Chinese value set. The experimental EQ-TIPS addresses HRQoL assessment for toddlers and infants. This study tested the psychometric properties of both instruments in paediatric COVID-19 patients, and compared the performance of self-complete and proxy EQ-5D-Y-3L.

**Methods:**

This longitudinal study recruited 861 COVID-19 patients aged 0–18 years and their parental caregivers, with 311 dyads completing the follow-up. Digital administration included the EQ-TIPS, the EQ-5D-Y-3L, and Overall Health Assessment (OHA). Controls comprised 231 healthy children. Analysis encompassed known-group validity, child-parent agreement, and responsiveness to change in disease severity and OHA.

**Results:**

COVID-19 children exhibited lower HRQoL than non-infected peers. The EQ-TIPS and the EQ-5D-Y-3L distinguished groups by disease presence, severity and symptoms, showing moderate to good known-group validity (ESs: 0.45–1.39 for EQ-TIPS, 0.44–1.91 for self-complete EQ-5D-Y-3L, and 0.32–1.67 for proxy EQ-5D-Y-3L). Child-parent agreement was moderate to good for EQ-5D-Y-3L (ICC: 0.653–0.823; Gwet’s AC1: 0.470–0.738), and responsiveness was good for both EQ-TIPS Level Sum Score (LSS) (ESs: 1.21–1.39) and EQ-5D-Y-3L index scores (ESs: 1.00–1.16).

**Conclusions:**

This study demonstrates the reliability, validity, and responsiveness of the experimental EQ-TIPS and the EQ-5D-Y-3L in paediatric COVID-19 patients. It is the first evidence of the EQ-TIPS’ responsiveness, supporting its use in assessing the impact of COVID-19 on paediatric HRQoL.

## Highlights

The study validates the experimental version of EQ-TIPS, offering the first evidence of EQ-TIPS’ responsiveness. It addresses the scarcity of validated instruments for infants and toddlers, contributing to improved paediatric HRQoL assessment.

The research pioneers the application of the Chinese value set for EQ-5D-Y-3L in paediatric COVID-19 patients. The study enhances the existing knowledge base by providing psychometric evidence for this instrument in a clinical context, supporting its utility in diverse healthcare settings.

Both instruments can be used in COVID-related clinical and resource allocation decision-making and in monitoring the well-being of infants, children and adolescents affected by COVID-19 and respiratory infections.

## Introduction

As of April 30, 2023, the World Health Organization (WHO) has reported over 765 million confirmed COVID-19 cases and 6.9 million deaths worldwide [1]. Beyond pulmonary complications and deaths, COVID-19 can impact the physical, emotional, and social well-being of children and adolescents, with increased rates of anxiety and depressive symptoms observed from ages 7 to 17 [2]. Longitudinal studies have indicated that COVID-19 impacts on physical and school-related aspects in adolescents aged ≥ 14 years [3]. Notably, research focusing on the vulnerable age group of birth to three years is scarce, despite their developmental stage and reliance on carers. A single study of infants and toddlers with atopic dermatitis found significantly worse HRQoL during the pandemic, underscoring the need for further research in this age group [4]. Furthermore, recruiting a population with COVID-19 not only offers insights into the direct effects of the disease but also presents a unique opportunity to study a diverse range of symptom severities among younger children, thus providing valuable data to understand the full spectrum of the pandemic’s impact on paediatric populations. However, existing generic HRQoL instruments which have been used to assess HRQoL in children and adolescents affected by COVID-19, such as KIDSCREEN-10, PedsQL 4.0, and KINDL-R questionnaire [5–7], lack societal preference-based scores which can be used in economic evaluations.

The EQ-5D-Y-3L is a widely used quality of life instrument for children and adolescents [8], which comes with a preference-based measures index score that can be used to calculate the Quality-Adjusted Life Years (QALYs). This provides insights into healthcare resource utilization and costs related to children’s HRQoL impact. There are different administration versions of EQ-5D-Y-3L, the self-complete and the proxy version. The ‘proxy version’ is crucial for parental evaluation when children cannot self-rate. For children aged 4–7 years, a proxy version should be used. In children aged over eight years, the self-complete version is generally recommended [9]. Despite demonstrating good reliability, validity, and responsiveness in paediatric patients with severe pneumonia and other respiratory conditions [10, 11], it lacks specific psychometric assessment for younger populations affected by COVID-19. Efforts in developing the Chinese EQ-5D-Y-3L value sets demand evaluation [12], especially for known-group validity and responsiveness in head-to-head studies [13–15]. Moreover, Studies indicate parents of children with COVID-19 or other infections may underestimate their child’s HRQoL [10, 16], underscore the importance of examining agreement and discordance between self-complete and proxy versions [9].

The experimental version of EQ-TIPS (EQ Toddler and Infant populations questionnaire), developed in 2018, assesses the physical, mental, emotional, and social functions of children aged 0 to 36 months [17]. Although currently in the experimental phase with no definitive version or available value sets, the EQ-TIPS has shown good construct validity in young children who have undergone general surgery, burn injury, or cardiac surgery [18]. However, additional research is needed to explore other properties, including reliability (examined in a small sample of the general population) [19], feasibility, and clinical utility. This research will be useful in moving the experimental EQ-TIPS towards an approved version, particularly with regard to cross-cultural validity.

During the COVID-19 pandemic in China, an opportunity arose to test the psychometric properties of the EQ-5D-Y-3L and the experimental EQ-TIPS in paediatric patients with this condition, utilizing the newly published Chinese value sets with the Y-3L. Therefore, this study had three objectives. First, to assess the validity, inter-rater reliability, and responsiveness of the self-complete version of the EQ-5D-Y-3L in children and adolescents aged 4–18 years. Second, to compare outcomes between the self-complete and proxy versions of the EQ-5D-Y-3L in those aged 6–18 years. Finally, to evaluate the distributional properties, known-group validity, and responsiveness of the experimental EQ-TIPS in children with COVID-19 aged under four years.

## Methods

### Sampling

This was a descriptive, longitudinal, prospective study with a repeated measures designed to test for reliability, validity and responsiveness of the instruments. We recruited paediatric inpatients and outpatients with confirmed COVID-19 infection and treated at Renji Academic Hospital in Shanghai from May 2022 to January 2023, along with their parental carers. A control group, consisting of infants, children and adolescents testing negative for COVID-19 tests with no related symptoms, was recruited using a ‘snowball approach’, primarily by reaching out to the siblings and friends of the patients.

For paediatric patients, the inclusion criteria were: (1) aged between 0 and 18 years; (2) confirmed COVID-19 infection through PCR (Polymerase Chain Reaction) or antigen test; (3) newly diagnosed by a specialist within the past month, without prior infection; and (4) admitted as inpatients or receiving outpatient care. Individuals aged 6–18 years, proficient in Chinese, and capable of independent questionnaire completion were eligible for the self-complete version. Those with other known respiratory viral infections within the preceding three months or known chronic health conditions were excluded.

For non-infected children and adolescents in the control group, the inclusion criteria were as follows: aged 0–18 years, no history of confirmed COVID-19 infection based on negative PCR or antigen test results, and generally healthy with no illnesses or symptoms suggestive of COVID-19 in the past three months. Exclusions applied to individuals not well enough to complete surveys or lacking written informed consent from legal guardians.

For carers, inclusion criteria were: (1) a primary carer was present in the week before the survey for the eligible child, (2) parent of an eligible child respondent, (3) physically present during the outpatient visit or admission, and (4) cognitively able to complete the surveys. The study received approval from the institutional medical ethical review board of Guizhou Medical University (Approval number: GMU2022303).

### Instruments

The EQ-5D-Y-3L assesses HRQoL with five dimensions (mobility; looking after myself; doing usual activities; having pain or discomfort; and feeling worried, sad, or unhappy) and three severity levels. Each health state in the EQ-5D-Y-3L can be summarized using level descriptors, generating 243 (3^5^) unique states. The best state, 11,111, indicates ‘no problems’ in any dimension, while the worst state, 33,333, indicates ‘a lot of problems’ in all dimensions. An index score of 1.0 represent the value of full health, and a score of 0.0 the value of death. Negative values represent health states with values below the value of death. The collection of index scores for all possible states is called a ‘value set’. It includes a 20-cm visual analogue scale (EQ VAS) for overall health rating [20]. We used proxy version 1 in this study, involving caregivers providing their impression of the child’s health on the survey day [9].

The experimental version of EQ-TIPS, completed by the primary caregiver or parent, assesses six dimensions: movement; play; pain; social interaction; communication; and eating. Like the EQ-5D-Y-3L, each dimension has three severity levels, forming a 6-digit code with 729 (3^6^) unique health states. The best state is 111,111. The EQ VAS is also included. In this study, the EQ-TIPS assessed HRQoL for children aged under four years old, as the EQ-5D-Y-3L proxy version is recommended for those aged four years and older [9].

The Chinese versions of EQ-5D-Y-3L and the experimental EQ-TIPS underwent translation per EuroQol Group guidelines [21]. Observations from previous surveys revealed a tendency for respondents to omit the impact of COVID-19, likely due to fluctuating conditions in many patients. Therefore, we proposed slight modifications to the instructions of the EQ-5D-Y-3L and the EQ-TIPS. Specifically, we added a short phrase before the original instructions as follows: (1) For the baseline survey completed by proxies: In comparison to the situation before the outbreak of the pandemic, please tick the ONE box that you think best describes the child’s health TODAY; (2) For the follow-up survey completed by proxies: In comparison to the situation during the outbreak of the pandemic, please tick the ONE box that you think best describes the child’s health TODAY; (3) For the self-completed version of EQ-5D-Y-3L: Taking into account the impact of the coronavirus pandemic, please tick the ONE box that you think best describes your health TODAY. The modification was approved by the EuroQol Research Foundation for use in the current study.

The Overall Health Assessment question (OHA), a valid measure of subjective health in children and adolescents [22], was phrased as ‘How is your overall health today? Is it excellent, good, fair, poor, or very poor?’ The proxy version gathered the caregiver’s impression of the patient’s overall health on the survey day.

The Chinese COVID-19 severity criteria were: (1) Mild: respiratory symptoms and fever; (2) Moderate: persistent high fever, cough, shortness of breath, with pneumonia imaging; (3) Severe: includes indicators such as high fever, tachypnoea, low oxygen saturation, respiratory distress, altered consciousness, and feeding difficulties [23].

Clinical recovery from COVID-19 was defined as having normal body temperature for over 3 days; mostly disappeared or significantly improved symptoms; significant absorption of pneumonia lesions on follow-up CT scan (if present); and either two consecutive negative RT-PCR tests, RT-PCR cycle threshold value ≥ 35, or three consecutive negative antigen tests [23].

### Procedures

**Fig. 1:**
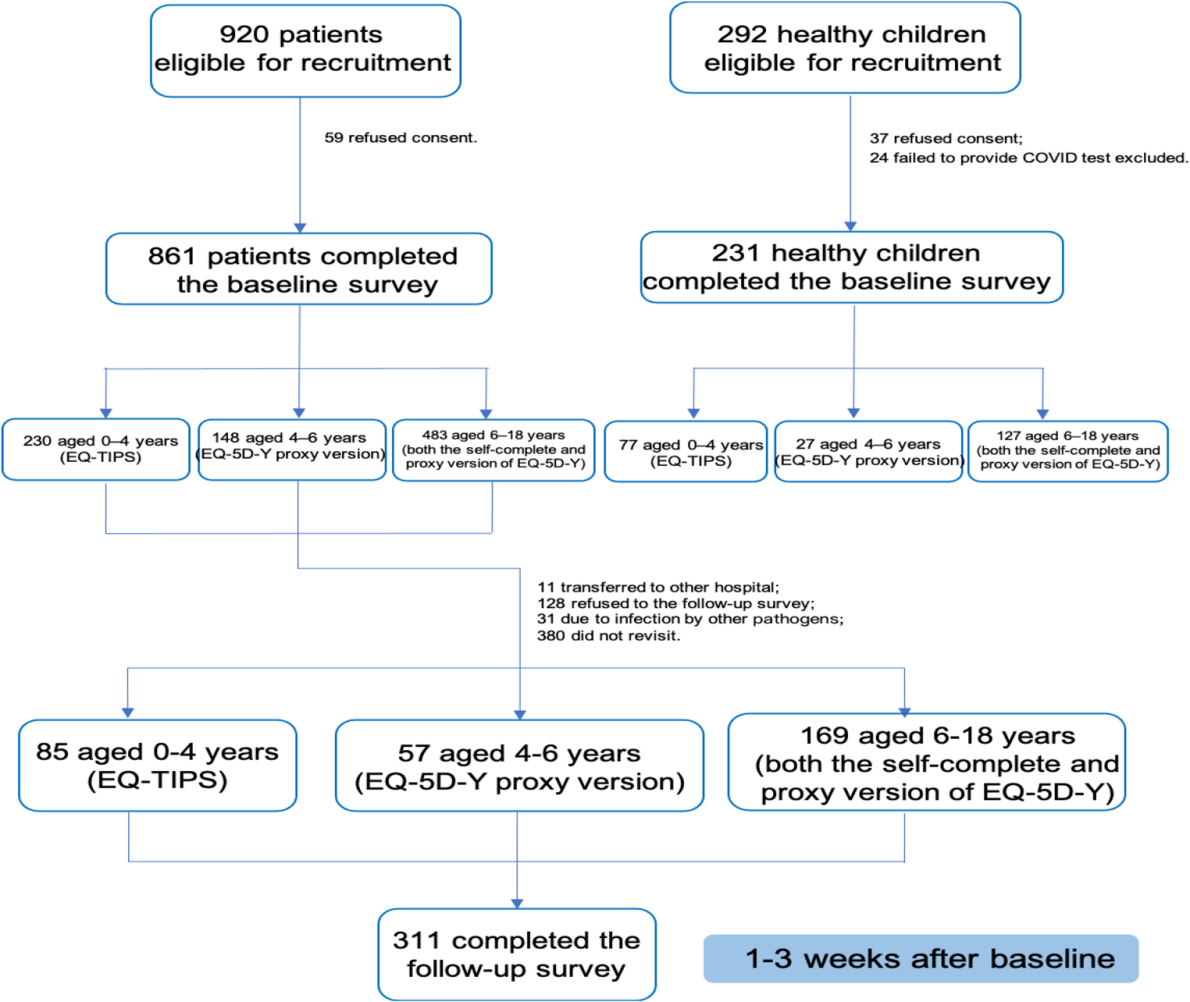
Flow chart of the study from recruitment of children and their parent carers

All consenting patients and parents independently completed the baseline survey using tablets in clinics or wards on the hospital admission day or during outpatient visits. Healthy children and adolescents, along with their parents, completed the survey at home using a smartphone. For children aged 4–18 years, parental carers provided sociodemographic information and completed the EQ-5D-Y-3L questionnaire (digital proxy version, including EQ-VAS), a five-point overall health assessment (OHA) question (proxy version), and questions on the parent’s demographics and the latest COVID-19 test result. For children under four years, the EQ-TIPS was used instead of the EQ-5D-Y-3L (Fig. 1).

The survey for children and adolescents aged over six years included the digital self-complete version of the EQ-5D-Y-3L, EQ VAS, and OHA question. Participants were invited to complete the same questionnaire during follow-up visits to outpatient clinics or on the day of discharge. Follow-up survey forms mirrored baseline forms, excluding demographic questions. On the survey day, the patient’s attending clinician completed the medical record including COVID-19 manifestations, disease duration, complications, severity per Chinese COVID-19 guidelines [23], and treatment.

### Data analysis

We calculated descriptive statistics to summarize demographic, socioeconomic, and clinical characteristics. The construct validity, inter-rater agreement, and responsiveness of the EQ-TIPS and the EQ-5D-Y-3L dimensions and summary scores (EQ index score, level sum score and EQ VAS) were assessed.

For EQ-5D-Y-3L: the index score and EQ VAS were generated separately for self-complete (≥ six years) and proxy versions, using the Chinese EQ-5D-Y-3L value set [12]. The index score ranges from − 0.088 to 1, with higher values indicating better health utility.

For EQ-TIPS: as no preference-based scoring is available, a level sum score (LSS) was employed to summarize responses on the descriptive system. Numeric values ranged from 6 (no problems on all six dimensions: 1 + 1 + 1 + 1 + 1 + 1 = 6) to 18 (most severe score: level 3 on all dimensions: 3 + 3 + 3 + 3 + 3 + 3 = 18) [24].

We evaluated known-group validity by comparing summary scores across four health status categories at baseline: 1) with or without COVID-19; 2) three grades of disease severity of COVID-19 (mild, moderate, or severe); 3) presence of two or more symptoms versus none or one symptom. Appendix Table 1 provides a detailed breakdown of the symptoms observed in our study population, allowing for a clearer understanding of how symptom presence correlates with disease severity; and 4) ‘excellent’/’good’ versus ‘fair’/’poor’/’very poor’ oral health assessment (OHA). Our hypothesis predicted higher EQ-5D-Y-3L index scores and EQ VAS, as well as lower EQ-TIPS LSS, in ‘good’ health groups compared to ‘poor’ health groups. We used independent t-tests, and ANOVA for comparisons, with Cohen’s D effect size (ES = difference of mean/ pooled SD) indicating the relative efficiency in discriminating between patients with different health conditions [25]. Individual dimension-level distribution analysis employed Chi-square test, and Fisher’s exact test if any cell had expected count less than 5.

**Table 1:** Descriptive statistics of the sample (*n* = 1092)

	EQ-TIPS sample					EQ-5D-Y-3L sample				
	COVID-19 *n* = 230		Non-infected *n* = 77		*p* value	COVID-19 *n* = 631		Non-infected *n* = 154		*p* value
*Children*										
*Age (month/year)*,* mean (SD)*	2.2	(1.1)	2.0	(1.1)	0.315	8.7	(3.3)	8.8	(2.9)	0.561
0-12mo (EQ-TIPS)/4–5 year (EQ-5D-Y-3L), % (n)	19.6	(45)	22.1	(17)	0.086	23.4	(148)	17.5	(27)	0.197
13-24mo (EQ-TIPS)/6–11 year (EQ-5D-Y-3L), % (n)	17.0	(39)	27.3	(21)		60.1	(379)	64.9	(100)	
25-48mo (EQ-TIPS)/12–18 year (EQ-5D-Y-3L), % (n)	63.5	(146)	50.6	(39)		16.5	(104)	17.5	(27)	
*Sex*,* % (n)*										
Male	52.6	(121)	53.2	(41)	0.923	52.8	(333)	57.8	(89)	0.263
Female	47.4	(109)	46.8	(36)		47.2	(298)	42.2	(65)	
*Disease duration*	10.0	(9.8)	/	/		11.6	(10.6)	/	/	
*Numbers of symptoms*										
0/1	20.4	(47)	/	/		22.7	(143)	/	/	
More than 2	79.6	(183)	/	/		77.3	(488)	/	/	
*Disease severity*										
Mild	32.2	(74)	/	/		37.6	(237)	/	/	
Moderate	43.9	(101)	/	/		45.6	(288)	/	/	
Severe	23.9	(55)	/	/		16.8	(106)	/	/	
*Proxy OHA*,* % (n)*										
1	14.3	(33)	24.7	(19)	0.002	18.4	(116)	29.9	(46)	< 0.001
2	34.3	(79)	29.9	(23)		29.2	(184)	33.8	(52)	
3	24.3	(56)	36.4	(28)		25.4	(160)	26.0	(40)	
4	21.3	(49)	7.8	(6)		20.8	(131)	9.1	(14)	
5	5.7	(13)	1.3	(1)		6.3	(40)	1.3	(2)	
*Self-report OHA (≥ 6 years)*,* % (n)*						*n* = 446		*n* = 120		
1						23.8	(106)	41.7	(50)	< 0.001
2						28.7	(128)	33.3	(40)	
3						20.2	(90)	17.5	(21)	
4						20.0	(89)	6.7	(8)	
5						7.4	(33)	0.8	(1)	
*Parent carer*										
*Age*,* mean (SD)*	32.0	(4.1)	31.9	(4.6)	0.957	37.5	(5.4)	37.0	(4.8)	0.342
*Residence*,* % (n)*										
Urban	92.2	(212)	89.6	(69)	0.098	94.5	(596)	85.1	(131)	< 0.001
Rural	5.7	(13)	10.4	(8)		4.8	(30)	14.9	(23)	
Refuse to answer	1.6	(5)	0.0	(0)		0.6	(5)	0.0	(0)	
*Relationship*										
Father	36.1	(83)	28.6	(22)	0.229	29.0	(183)	26.0	(40)	0.455
Mother	63.9	(147)	71.4	(55)		71.0	(448)	74.0	(114)	
*Education*,* % (n)*										
Middle/high school	7.4	(17)	6.5	(5)	0.493	7.7	(48)	5.2	(8)	0.367
College or above	92.2	(212)	93.5	(72)		92.3	(577)	94.8	(145)	
Refuse to answer	0.4	(1)	0.0	(0)		1.0	(6)	0.6	(1)	

SD – standard deviation; OHA – overall health assessment

Inter-rater reliability was evaluated between patients and carers on the EQ-5D-Y-3L at baseline. For dimensions, Gwet’s AC1 assessed agreement [26], with values categorized as: below 0.2 (poor), 0.21–0.4 (fair), 0.41–0.6 (moderate), 0.61–0.8 (good), and above 0.8 (excellent) [27]. The agreement on index score and EQ VAS used the intraclass correlation coefficient (ICC), with values classified as: below 0.1 (no agreement), 0.1–0.29 (low agreement), 0.3–0.49 (moderate agreement), 0.5 or higher (high agreement), and 0.7 or above (good reliability) [28].

Responsiveness was assessed in patients showing clinical recovery or OHA improvement from baseline to follow-up via independent t-tests to compare mean summary scores. Changes in ‘no problem’ proportions for each dimension were analysed. The results include the Glass’ Δ effect size (ES = difference of mean/ baseline SD), which is recommended when the intervention might influence the standard deviation [29]. The percentage of ‘no problem’ reported are detailed in Appendix-Table 3. All analyses utilized SPSS (IBM SPSS Statistics, Version 26.0, IBM Corp).

## Results

Figure 1 illustrates the recruitment of this study involving 1092 children (0–18 years) and their parental caregivers. Among them, 78.8% were COVID-19 infected (average duration: 10.9 days), with 311 completing the follow-up survey after one to three weeks. The control group (21.2%) comprised non-infected children staying at home for at least three months. Baseline characteristics presented in Table 1 showed no significant differences except in residence. Most EQ-TIPS respondents were aged 2–4 years (63.5%), while the EQ-5D-Y-3L completers were mostly 6–11 years old (61.0%). Approximately 80% of patients had at least two symptoms, two-thirds had moderate to severe disease, and most caregivers were highly educated (92.1%).

**Fig. 2:**
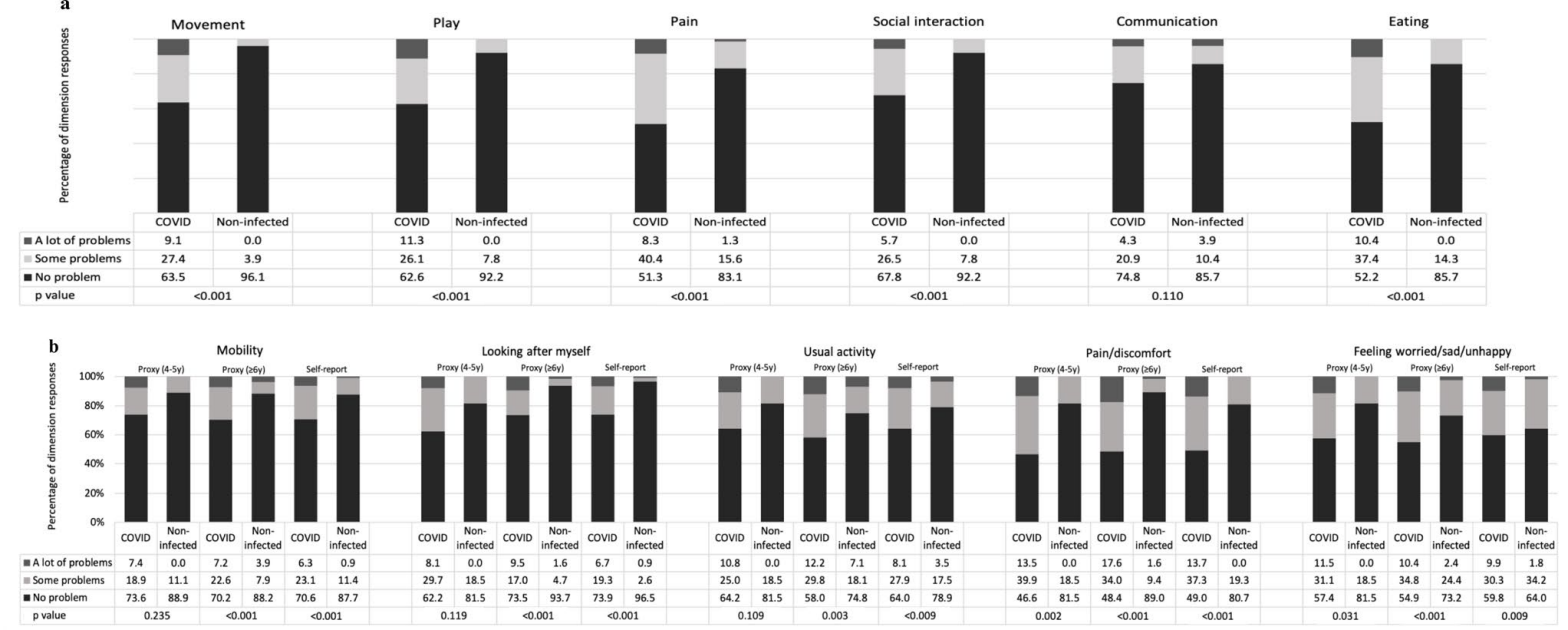
Dimension responses of **the EQ-TIPS** and **the EQ-5D-Y-3L** for children with or without COVID-19 infection in different age groups, i.e., aged 0–3 years, 4–5 years, and ≥ 6 years. P-value represent differences between with children COVID-19 and a healthy sample in terms of Chi-square test, and Fisher’s exact test if any cell had expected count less than 5. (**a**) Percentage of dimension responses for the EQ-TIPS for patients with COVID-19 and children without infection aged 0–3 years. (**b**) Percentage of item responses for the EQ-5D-Y-3L for patients with COVID-19 and children without infection aged ≥ four years

Figure 2a shows that all EQ-TIPS dimensions contribute to lower scores, with parents of non-infected children reporting fewer problems than those with COVID-19 (*p* < 0.001), except for ‘communication’ (*p* = 0.110). The proportion reporting ‘no problems’ ranged from 51.3% for ‘pain’ to 74.8% for ‘communication’. Full health (111111) was reported by 30.4% of patients and 61.0% of non-infected children, indicating a higher ceiling effect in the healthier non-infected group across all dimensions as expected, ranging from 83.1% for ‘pain’ to 96.1% for ‘movement’.

Figure 2b indicates a significant trend of reporting more problems by infected children aged ≥ 4 years on the EQ-5D-Y-3L. In the self-complete version, the proportion of patients reporting ‘no problems’ ranged from 49.0% for ‘pain/discomfort’ to 73.9% for ‘looking after myself’. The proxy version showed a similar pattern with slightly more problems reported, particularly at the extreme level. Full health (11111) was reported by 38.4% of patients using the self-complete version, and by 56.1% of non-infected children. The proxy version percentages were 36.0% for patients and 53.5% for non-infected children. In patients aged 4–5 years, physical items (‘mobility’, ‘looking after myself’ and ‘usual activity’) reported less problems, with no significant difference compared to the non-infected group (p = 0.235, 0.119, and 0.109, respectively).

Table 2 presents the known-group validity of EQ-TIPS and EQ-5D-Y-3L summary scores. Those with poorer health status—COVID-19 infection, higher disease severity, multiple symptoms, or poorer OHA—showed higher LSS, lower index and EQ VAS scores. Statistically significant differences were observed between relevant groups (*p* < 0.05), except for EQ-TIPS LSS between mild and moderate severity (absolute difference = 0.01). Cohen’s D ESs were mostly moderate to high. For the EQ-TIPS LSS, between-groups ESs ranged from 0.58 to 0.84, and for the EQ-5D-Y-3L index, from 0.32 to 0.65 in those aged 4–18 years. ESs of the self-complete EQ-5D-Y-3L index score were larger than proxy version in ages ≥ 6 years (0.44 to 1.26 vs. 0.32 to 0.76). These differences in ESs were particularly evident when categorized based on disease severity and OHA. The greatest discriminative ability, with large effect sizes (0.76 to 1.54), was observed between OHA-defined groups, in all age groups. Satisfactory discriminative validity was shown between the COVID-19 and symptom-based groups (ESs: 0.50 to 0.84 and 0.60 to 0.76, respectively). The EQ-TIPS and the EQ-5D-Y-3L tended to show larger ESs between patients in the moderate and severe groups, compared to the differences between the mild and moderate groups.

**Table 2:** Known-groups validity of the EQ-TIPS LSS, the EQ-5D-Y-3L index score, and EQ VAS (mean [SD]) across different health condition based on with or without COVID-19 infection, disease severity, number of symptoms, and OHA using t-test or ANOVA

	With or without COVID-19			Disease severity					Number of symptoms			OHA		
	Non-infected	Infected	Cohen’s d ES (95%CI)	Mild	Moderate	Severe	Cohen’s d ES (95%CI) Mild vs. Moderate	Cohen’s d ES (95%CI) Moderate vs. Severe	No/single	Multiple	Cohen’s d ES (95%CI)	Very good/ Good	Fair/Poor/ Very Poor	Cohen’s d ES (95%CI)
*0–3 years (proxy)*	*n* = 77	*n* = 230		*n* = 74	*n* = 101	*n* = 55			*n* = 47	*n* = 183		*n* = 112	*n* = 118	
EQ-TIPS LSS	6.70 (1.12)	8.77 (2.78)	0.84 (0.57, 1.10)	8.39 (2.70)	8.40 (2.60)	9.96 (2.91)	0.004 ^§^ (-0.29, -0.30)	0.58 (0.24, 0.91)	7.49 (2.09)	9.10 (2.84)	0.60 (0.27, 0.92)	7.69 (2.34)	9.80 (2.78)	0.82 (0.55, 1.09)
EQ VAS	85.5 (13.4)	67.4 (22.8)	-0.86 (-1.10, -0.27)	72.5 (21.1)	68.6 (23.3)	58.4 (21.9)	-0.17 ^§^ (-0.48, -0.13)	-0.45 (-0.78, -0.11)	78.0 (17.5)	64.7 (23.3)	-0.60 (-0.92, -0.27)	80.8 (14.3)	54.7 (22.2)	-1.39 (-1.68, -1.10)
*4–5 years (proxy)*	*n* = 27	*n* = 148		*n* = 50	*n* = 60	*n* = 38			*n* = 30	*n* = 118		*n* = 78	*n* = 70	
EQ-5D-Y-3L index	0.95 (0.06)	0.74 (0.36)	-0.63 (-1.05, -0.22)	0.91 (0.17)	0.73 (0.34)	0.54 (0.44)	-0.65 (-1.04, -0.27)	-0.50 (-0.91, -0.09)	0.95 (0.12)	0.69 (0.38)	-0.76 (-1.16, -0.34)	0.99 (0.16)	0.53 (0.40)	-1.54 (-1.91, -1.17)
EQ VAS	87.2 (10.0)	70.3 (26.5)	-0.68 (-1.10, -0.27)	84.7 (16.0)	66.4 (28.7)	57.5 (25.8)	-0.77 (-1.16, -0.38)	-0.32 ^§^ (-0.71, -0.09)	87.9 (13.3)	65.8 (27.2)	-0.88 (-1.30, -0.47)	86.4 (14.4)	52.4 (25.4)	-1.67 (-2.04, -1.30)
*6–18 years*														
*Self-report*	*n* = 114	*n* = 445		*n* = 172	*n* = 214	*n* = 59			*n* = 102	*n* = 343		*n* = 233	*n* = 212	
EQ-5D-Y-3L index	0.92 (0.13)	0.77 (0.33)	-0.50 (-0.71, -0.29)	0.87 (0.23)	0.74 (0.34)	0.57 (0.41)	-0.44 (-0.64, -0.24)	-0.48 (-0.77, -0.19)	0.92 (0.19)	0.72 (0.35)	-0.62 (-0.85, -0.34)	0.94 (0.15)	0.59 (0.37)	-1.26 (-1.46, -1.06)
EQ VAS	86.5 (12.6)	72.2 (25.0)	-0.62 (-0.83, -0.41)	80.9 (19.9)	68.8 (26.4)	52.9 (24.9)	-0.51 (-0.71, -0.31)	-0.61 (-0.90, -0.32)	85.4 (17.0)	68.3 (25.6)	-0.72 (-0.94, -0.49)	88.7 (11.4)	54.1 (23.3)	-1.91 (-2.14, -1.69)
*Proxy-report*	*n* = 127	*n* = 483		*n* = 187	*n* = 228	*n* = 68			*n* = 112	*n* = 371		*n* = 222	*n* = 261	
EQ-5D-Y-3L index	0.91 (0.19)	0.72 (0.37)	-0.56 (-0.76, -0.36)	0.81 (0.31)	0.70 (0.37)	0.52 (0.41)	-0.32 (-0.51, -0.12)	-0.47 (-0.75, -0.20)	0.90 (0.21)	0.66 (0.39)	-0.67 (-0.89, -0.46)	0.86 (0.29)	0.60 (0.38)	-0.76 (-0.95, -0.58)
EQ VAS	87.8 (10.9)	69.9 (24.9)	-0.79 (-0.99, 0.59)	78.6 (20.6)	67.8 (24.6)	56.9 (24.3)	-0.47 (-0.67, -0.28)	-0.44 (-0.72, 0.17)	84.4 (17.6)	65.3(25.1)	-0.81 (-1.03, -0.59)	85.3 (15.8)	56.8 (23.7)	-1.39 (-1.59, -1.19)

§: p>0.05; all other p values were >0.01. ES – effect size; LSS – level sum score; VAS – visual analogue scale; OHA – overall health assessment

The EQ VAS exhibited moderate to high known-group validity for both EQ-TIPS and EQ-5D-Y-3L, with larger effect sizes observed in older age groups (0.17 to 1.39 for 0–3 years, 0.32 to 1.67 for 4–5 years, 0.44 to 1.91 for 6–18 years, respectively). Additionally, the self-complete version (0.51 to 1.91) showed higher ESs compared to the proxy version (0.44 to1.39).

Table 3 presents the inter-rater agreement on EQ-5D-Y-3L dimensions using data from 445 patient-proxy dyads with COVID-19 and a total of 559 child-parent dyads at baseline. For patients with COVID-19, the Gwet’s AC1 values ranged from 0.470 for ‘having pain/discomfort’ to 0.687 for ‘mobility’, demonstrating moderate to good inter-rater reliability for the descriptive system. The ICC values for index and EQ VAS were 0.657 and 0.815, respectively, indicating good inter-rater reliability for both. The overall sample exhibited similar and slightly better reliability, with Gwet’s AC1 ranging from 0.529 to 0.738 and 0.653 to 0.823 for ICC.

**Table 3:** The child-parent agreement of the self-complete and proxy versions of the EQ-5D-Y-3L at baseline in children ≥ 6 years and their parent carers (*n* = 559)

	Patients with COVID-19 *n* = 445			Overall sample *n* = 559		
	Gwet’s AC1	95% CI	Agreement(%)	Gwet’s AC1	95% CI	Agreement(%)
Mobility (Walking about)	0.687	0.631, 0.743	75.7	0.703	0.665, 0.751	76.4
Looking after myself	0.685	0.630, 0.741	75.1	0.738	0.692, 0.783	78.5
Doing usual activities	0.613	0.552, 0.674	71.7	0.625	0.572, 0.579	72.1
Having pain/ discomfort	0.470	0.403, 0.536	63.1	0.529	0.472, 0.587	66.4
Feeling worried/ sad/unhappy	0.552	0.488, 0.616	67.6	0.560	0.504, 0.616	67.8
	ICC	95% CI	*p* value	ICC	95% CI	*p* value
EQ-5D-Y-3L index	0.657	0.601, 0.707	< 0.001	0.653	0.603, 0.698	< 0.001
EQ VAS	0.815	0.782, 0.844	< 0.001	0.823	0.795, 0.848	< 0.001

VAS – visual analogue scale

Table 4 shows strong responsiveness of the EQ-TIPS and the EQ-5D-Y-3L for both groups to health improvement based on clinical progress and enhanced Overall Health Assessment (OHA). The EQ-TIPS LSS showed ES of 1.21–1.39, and the EQ-5D-Y-3L index score had ES of 1.00–1.16 and 1.08–1.15 for the proxy and self-complete versions, respectively, in children and adolescents with improved health. The EQ VAS demonstrated the highest responsiveness, with SES ranging from 1.38 to 2.01 for proxy versions and 1.77 to 1.94 for self-complete version.

**Table 4:** Change in mean (SD) of EQ-TIPS LSS, EQ-5D-Y-3L index score and EQ VAS (with corresponding effect sizes) between illness and recovery based on clinical recovery or improved OHA

	COVID-19 recovery			Improved OHA		
	Baseline	Follow-up	Glass’ Δ ES	Baseline	Follow-up	Glass’ Δ ES
*0–3 years (proxy)*		*n* = 69			*n* = 64	
EQ-TIPS LSS, mean (SD)	10.33 (2.80)	6.93 (1.83)	1.21	10.34 (2.61)	6.70 (1.29)	1.39
EQ VAS, mean (SD)	51.6 (21.6)	85.2 (16.7)	-1.56	48.4 (21.4)	85.8 (13.3)	-1.75
*4–5 years (proxy)*		*n* = 48			*n* = 46	
EQ-5D-Y-3L index, mean (SD)	0.47 (0.44)	0.91 (0.17)	-1.00	0.45 (0.43)	0.90 (0.17)	-1.05
EQ VAS, mean (SD)	48.7 (27.4)	86.5 (9.5)	-1.38	45.1 (24.2)	84.5 (10.9)	-1.63
*6–18 years*						
*Self-report*		*n* = 132			*n* = 138	
EQ-5D-Y-3L index, mean (SD)	0.51 (0.38)	0.92 (0.21)	-1.08	0.48 (0.39)	0.93 (0.19)	-1.15
EQ VAS, mean (SD)	47.4 (23.1)	88.4 (11.0)	-1.77	45.5 (21.8)	87.9 (10.8)	-1.94
*Proxy-report*		*n* = 141			*n* = 147	
EQ-5D-Y-3L index, mean (SD)	0.45 (0.41)	0.91 (0.22)	-1.12	0.44 (0.41)	0.91 (0.20)	-1.16
EQ VAS, mean (SD)	46.8 (20.8)	87.6 (11.3)	-1.96	45.8 (20.7)	87.5 (11.2)	-2.01

All *p* value >0.01 using independent t-tests; SD – standard deviation; OHA – overall health assessment; VAS – visual analogue scale; ES – effect size

## Discussion

In this study, we observed acceptable psychometric properties for the Chinese versions of the experimental EQ-TIPS, and for both modes of administration (self-complete and proxy) for the EQ-5D-Y-3L. These findings add to an expanding evidence base for the psychometric robustness of the EQ-5D-Y-3L and provide the first evidence of responsiveness for the EQ-TIPS, which is one of only a handful of preference-weighted HRQoL measures that can be used in the youngest populations. The lack of validated preference-weighted measures for infants and toddlers means that the EQ-TIPS is likely to be widely used, which highlights the importance of providing evidence to support its psychometric performance. Notably, large effect sizes on the EQ-5D-Y-3L were observed when using both clinical changes (disease severity and symptom numbers) and self-rated health changes (OHA) as external criteria for assessing responsiveness (independently of whether proxy or self-report was used), suggesting that the EQ-5D-Y-3L is useful in capturing improvements in health after recovery from COVID-19.

Our study, characterized by a large and diverse sample, broad age representation, and the unique ability to assess responsiveness through clinical recovery criteria, has several strengths. Notably, this is the first study in China to assess the psychometric performance of the experimental EQ-TIPS. Only three studies have been published previously exploring the EQ-TIPS’ measurement properties. While they demonstrated its validity, they provided limited evidence of reliability and none for responsiveness [18, 19, 30]. Our research therefore contributes the first evidence of the EQ-TIPS’ responsiveness, indicating its suitability for capturing COVID-19-related HRQoL improvements in infants and toddlers. Additionally, our study is the first to examine the psychometric properties of the EQ-5D-Y-3L in patients with COVID-19 using the corresponding Chinese value set.

The EQ-TIPS, applied to COVID-19 patients, showed a ceiling effect in the ‘communication’ dimension, echoing findings in a broader paediatric health study [30]. The non-significant difference suggests minimal impact on children’s communicative abilities. Respiratory effects might not significantly affect physical communication skills in young children. Our previous cognitive interviews revealed parental difficulty in responding to this dimension, emphasizing the need for simplified examples. This aligns with the study’s highest ceiling effect (74.8%) in ‘communication’ among COVID-19-infected children.

The EQ-TIPS LSS discriminated effectively between infected and non-infected groups and individuals with varying OHA, exhibiting significant effect sizes. It also discriminated well between those with moderate and severe disease, with an effect size of 0.58, but not between those with mild and moderate disease (ES of 0.004). It is not clear why this should be the case, but it is of note that the EQ VAS also provided poorer results in this youngest group (ES of 0.17) compared to any of the other age groups when examining its ability to discriminate between patients classified as mild and moderate. As an identical EQ VAS is used in the EQ-TIPS as in the EQ-5D-Y-3L, this finding suggests that the lack of discriminatory capacity between mild and moderate disease is not necessarily due to the instrument itself but rather to other factors. These factors could include difficulties that parents have in deciding on an ‘accurate’ score for their child’s health in such young children, who are unable to communicate how they are feeling, and/or questions about whether the criteria used to decide on disease severity are equally suitable across all age groups. Further research is required to clarify these issues.

This study offers the first evidence of the EQ-TIPS’ responsiveness, revealing significant improvement across all dimensions, as shown by the change in the percentage of patients reporting ‘no problems’ from the first to the second visit. Improvements were observed on all dimensions, suggesting that each dimension serves as a useful indicator of how HRQoL evolves as COVID-19 symptoms improve over time in very young children.

Our study supported the known-group validity, inter-rater reliability, and responsiveness of both self-complete and proxy versions of the EQ-5D-Y-3L, as evidenced by the performance of the index scores and EQ VAS. While previous studies validating the proxy version have mainly compared agreement levels between children and proxy-respondents [13–15, 31–33], our study delved into the comparison of validity and responsiveness, in particular, an area with limited exploration [34, 35].

In general, the response distribution patterns were similar between the self-complete and proxy versions, regardless of infection status. Our findings align with previous studies, suggesting parental underestimation of children’s HRQoL, particularly in COVID-19 or other infections [10, 16]. Specifically, parents reported more problems at level 3, which could be attributed to parents perceiving children’s symptoms as severe, while children might exhibit greater physical tolerance and cope better with the illness [36]. Additionally, no high ceiling effects were observed in any EQ-5D-Y-3L dimensions for children or adolescents with COVID-19, indicating the effectiveness of capturing these variations and identifying HRQoL-related problems or limitations.

The EQ-5D-Y-3L demonstrates moderate to good discriminative ability across age groups and health categories, with its strongest performance observed in OHA, consistent with its generic scale nature. Although the instrument excels in capturing variations in symptoms and infection status, its capacity to discern subtleties in disease severity may be constrained by its generic design, especially in scenarios where the distinction between ‘mild’ and ‘moderate’ criteria is not substantial. Nonetheless, the statistical significant differences in index values between mild, moderate, and severe categories suggested that the EQ-5D-Y-3L is a sensitive instrument in COVID-19 economic evaluations. This is particularly relevant for interventions such as vaccines, which can prevent poorer health states, or for treatments that improve health. Additionally, the substantial similarities between self-reports and proxy results suggest that results in trials will be relatively comparable, whichever source is used to collect data on the EQ-5D-Y-3L descriptive system. Although proxies tended to score lower than self-report, the difference between categories of disease severity or number of symptoms is quite similar, so gains or losses will be similar whether self-report or proxy reports are used. For instance, moving from severe to moderate disease severity represents a move from 0.57 to 0.74 using self-report, and 0.52 to 0.70 for proxy, an almost identical difference, suggesting use of one or the other response mode would have little impact on in the context of an economic model.

Our study indicated good inter-rater reliability, supporting the idea that self-report and proxy data are likely to be relatively comparable, and that aggregating them, for example for use in an economic model, is likely to be acceptable. In our study, the reliability of the EQ-5D-Y-3L index score was good, and excellent for EQ VAS indicated by ICC. However, compared to physical items, ‘having pain or discomfort’ and ‘feeling worried, sad, or unhappy’ showed poorer child-parent agreement, with parents reported more problems and lower index scores and EQ VAS. This aligns with previous studies which found lower agreement for emotional and mental items in paediatric populations with haematological malignancies, idiopathic scoliosis or general population and their parents [13, 15, 37]. The impact of COVID-19 has further highlighted discrepancies across all dimensions, potentially influenced by factors such as parental education, household income, and the infection status of other family members [16]. For example, in our study, 44.6% of proxy respondents of children with COVID-19, reported recent personal infection within the past week.

To our knowledge, this study provides the largest sample for assessing the responsiveness of the EQ-5D-Y-3L in children with COVID-19, especially given the ability to generate index scores based on a recently published value set. Our findings demonstrated good responsiveness to clinical recovery from COVID-19 and health improvements based on overall health assessments (OHA). The considerable effect sizes observed for the EQ-5D-Y-3L index or LSS scores, along with notably larger SESs for EQ VAS, underscore the EQ-5D-Y-3L’s effectiveness in measuring health improvements. Moreover, the effective performance of both the experimental EQ-TIPS and the EQ-5D-Y-3L in children with COVID-19 implies their potential applicability to other prevalent respiratory infectious diseases, which are widespread in many countries. Although our study did not delve into intervention analysis, our future research will explore how specific COVID-19 interventions impact children’s HRQoL. Additionally, investigating whether the new EQ-5D-Y-5 L performs as well or better in these patients than the EQ-5D-Y-3L would be of interest [38].

This study has several limitations. Firstly, the data were collected at an academic hospital in Shanghai, involving participants with a relatively higher socioeconomic status and parental education background, limiting generalizability. Secondly, the instruction section of the EQ-TIPS and the EQ-5D-Y-3L was slightly modified to emphasize the impact by the COVID-19 pandemic, which could potentially affect responses when compared to use of the standard instruction.

## Conclusion

In conclusion, the study results show that the experimental EQ-TIPS and the EQ-5D-Y-3L are reliable and valid instruments for assessing the impact of COVID-19 on the HRQoL of children and adolescents, including very young children. Additionally, both instruments are responsive to change as children’s COVID-related health status evolves over time. The study also provides the first application of the new EQ-5D-Y-3L Chinese value set in a clinical population and shows that the values discriminate well between relevant disease groups. These instruments will therefore likely be useful in COVID-related clinical and resource allocation decision-making and in monitoring the well-being of infants, children and adolescents affected by COVID-19 and respiratory infections. Further research using these instruments to explore the impact of specific treatments for COVID-19 would be of interest.

## Appendix

**Appendix-Table 1:** COVID-19 severity and symptom distribution based on symptom numbers at baseline

	Number of symptoms *n*=861				
	No *n*=16	Single *n*=173	Multiple *n*=672	*p* value No vs. Single	*p* value No vs. Single vs. Multiple
*COVID-19 severity, %(* *n)*					
Mild	100.0 (16)	98.3 (170)	18.6 (125)	0.766	<0.001
Moderate	0.0 (0)	0.0 (0)	57.9 (389)		
Severe	0.0 (0)	1.7 (3)	23.5 (158)		
*Symptom*,* %(n)*					
Fever					
No	100.0 (16)	26.6 (46)	4.0 (27)	<0.001	<0.001
Yes	0.0 (0)	73.4 (127)	96.0 (645)		
Upper respiratory problems					
No	100.0 (16)	78.6 (136)	21.3 (143)	0.045	<0.001
Yes	0.0 (0)	21.4 (37)	78.7 (529)		
Pneumonia					
No	100.0 (16)	100.0 (173)	92.1 (619)	na	<0.001
Yes	0.0 (0)	0.0 (0)	7.9 (53)		
Pain					
No	100.0 (16)	98.3 (170)	60.9 (409)	0.817	<0.001
Yes	0.0 (0)	1.7 (3)	39.1 (263)		
Cardiovascular problems					
No	100.0 (16)	100.0 (173)	89.1 (599)	na	0.027
Yes	0.0 (0)	0.0 (0)	10.9 (73)		
Gastrointestinal problems					
No	100.0 (16)	98.8 (171)	70.8 (476)	0.904	<0.001
yes	0.0 (0)	1.2 (2)	29.2 (196)		
Fatigue					
No	100.0 (16)	96.0 (166)	51.3 (345)	0.541	<0.001
Yes	0.0 (0)	4.0 (7)	48.7 (327)		
Olfaction/ Gustation					
No	100.0 (16)	100.0 (173)	80.2 (539)	na	<0.001
Yes	0.0 (0)	0.0 (0)	19.8 (133)		
Shock					
No	100.0 (16)	100.0 (173)	93.9 (631)	na	0.038
Yes	0.0 (0)	0.0 (0)	6.1 (41)		
Eye problems					
No	100.0 (16)	100.0 (173)	94.2 (633)	na	0.048
Yes	0.0 (0)	0.0 (0)	5.8 (39)		

*P* value based on using Fisher’s exact test

**Appendix-Table 2:** Known-group’s validity: dimension-level response distributions and “no problem” reported for individual dimension across disease severity

	Mild % (*n*)			Moderate % (*n*)			Severe % (*n*)		
	Level 1	Level 2	Level 3	Level 1	Level 2	Level 3	Level 1	Level 2	Level 3
*0–3 years*	*n* = 74			*n* = 101			*n* = 55		
Movement, % (n)	66.2 (49)	23.0 (17)	10.8 (8)	70.3 (71)	24.8 (25)	5.0 (5)	47.3 (26)	38.2 (21)	14.5 (8)
Play, %	70.3 (52)	20.3 (15)	9.5 (7)	64.4 (65)	26.7 (27)	8.9 (9)	49.1 (27)	32.7 (18)	18.2 (10)
Pain, %	52.7 (39)	41.9 (31)	5.4 (4)	59.4 (60)	34.7 (35)	5.9 (6)	34.5 (19)	49.1 (27)	16.4 (9)
Social interaction, %	73.0 (54)	24.3 (18)	2.7 (2)	69.3 (70)	24.8 (25)	5.9 (6)	58.2 (32)	32.7 (18)	9.1 (5)
Communication, %	77.0 (57)	20.3 (15)	2.7 (2)	78.2 (79)	19.8 (20)	2.0 (2)	65.5 (36)	23.6 (13)	10.9 (6)
Eating, %	63.5 (47)	25.7 (19)	10.8 (8)	54.5 (55)	37.6 (38)	7.9 (8)	32.7 (18)	52.7 (29)	14.5 (8)
*4–5 years*	*n* = 50			*n* = 60			*n* = 38		
Mobility, %	90.0 (45)	8.0 (4)	2.0 (1)	76.7 (46)	20.0 (12)	3.3 (2)	47.4 (18)	31.6 (12)	21.1 (8)
Looking after myself, %	80.0 (40)	18.0 (9)	2.0 (1)	61.7 (37)	33.3 (20)	5.0 (3)	39.5 (15)	39.5 (15)	21.1 (8)
Usual activity, %	82.0 (41)	16.0 (8)	2.0 (1)	60.0 (36)	26.7 (16)	13.3 (8)	47.4 (18)	34.2 (13)	18.4 (7)
Pain/discomfort, %	72.0 (36)	24.0 (12)	4.0 (2)	40.0 (24)	45.0 (27)	15.0 (9)	23.7 (9)	52.6 (20)	23.7 (9)
Feeling worried/sad/unhappy, %	84.0 (42)	14.0 (7)	2.0 (1)	46.7 (28)	41.7 (25)	11.7 (7)	39.5 (15)	36.8 (14)	23.7 (9)
*6–18 years*									
*Self-report*	*n* = 172			*n* = 214			*n* = 59		
Mobility, %	84.3 (145)	12.8 (22)	2.9 (5)	65.0 (139)	27.1 (58)	7.9 (17)	50.8 (30)	39.0 (23)	10.2 (6)
Looking after myself, %	84.9 (146)	11.6 (20)	3.5 (6)	70.6 (151)	23.4 (50)	6.1 (13)	54.2 (32)	27.1 (16)	18.6 (11)
Usual activity, %	75.6 (130)	18.0 (31)	6.4 (11)	59.8 (128)	32.7 (70)	7.5 (16)	45.8 (27)	39.0 (23)	15.3 (9)
Pain/discomfort, %	65.7 (113)	30.2 (52)	4.1 (7)	41.1 (88)	43.0 (92)	15.9 (34)	28.8 (17)	37.3 (22)	33.9 (20)
Feeling worried/sad/unhappy, %	71.5 (123)	23.3 (40)	5.2 (9)	56.5 (121)	30.8 (66)	12.6 (27)	37.3 (22)	49.2 (29)	13.6 (8)
*Proxy*	*n* = 187			*n* = 228			*n* = 68		
Mobility, %	80.2 (150)	15.0 (28)	4.8 (9)	68.0 (155)	24.6 (56)	7.5 (17)	50.0 (34)	36.8 (25)	13.2 (9)
Looking after myself, %	80.7 (151)	12.8 (24)	6.4 (12)	73.7 (168)	18.0 (41)	8.3 (19)	52.9 (36)	25.0 (17)	22.1 (15)
Usual activity, %	70.1 (131)	20.3 (38)	9.6 (18)	54.8 (125)	34.6 (79)	10.5 (24)	35.3 (24)	39.7 (27)	25.0 (17)
Pain/discomfort, %	61.0 (114)	28.3 (53)	10.7 (20)	43.0 (98)	37.7 (86)	19.3 (44)	32.4 (22)	36.8 (25)	30.9 (21)
Feeling worried/sad/unhappy, %	67.9 (127)	24.6 (46)	7.5 (14)	49.6 (113)	39.9 (91)	10.5 (24)	36.8 (25)	45.6 (31)	17.6 (12)

**Appendix-Table 3:** Responsiveness: change in percentage of respondent reporting “no problem” for each dimension between illness and clinical recovery or improved OHA

	COVID recovery		Improved OHA	
	Baseline, % (*n*)	Follow-up, % (*n*)	Baseline, % (*n*)	Follow-up, % (*n*)
*0–3 years*		*n* = 69		*n* = 64
Movement, % (n)	37.7 (26)	88.4 (61)	37.5 (24)	93.8 (60)
Play, %	31.9 (22)	89.9 (62)	29.7 (19)	92.2 (59)
Pain, %	30.4 (21)	85.5 (59)	32.8 (21)	89.1 (57)
Social interaction, %	46.4 (32)	87.0 (60)	45.3 (29)	87.5 (56)
Communication, %	68.1 (47)	87.0 (60)	67.2 (43)	89.1 (57)
Eating, %	39.1 (27)	81.2 (56)	35.9 (23)	84.4 (54)
*4–5 years*		*n* = 48		*n* = 46
Mobility, %	56.3 (27)	91.7 (44)	50.0 (23)	89.1 (41)
Looking after myself, %	39.6 (19)	91.7 (44)	32.6 (15)	87.0 (40)
Usual activity, %	37.5 (18)	81.3 (39)	34.8 (16)	73.9 (34)
Pain/discomfort, %	16.7 (8)	81.3 (39)	13.0 (6)	78.3 (36)
Feeling worried/sad/unhappy, %	39.6 (19)	68.8 (33)	30.4 (14)	67.4 (31)
*6–18 years*				
*Self-report*		*n* = 132		*n* = 138
Mobility, %	40.2 (53)	90.9 (120)	37.7 (52)	91.3 (126)
Looking after myself, %	42.4 (56)	91.7 (121)	39.9 (55)	92.0 (127)
Usual activity, %	31.1 (41)	88.6 (117)	29.0 (40)	89.9 (124)
Pain/discomfort, %	15.9 (21)	80.3 (106)	14.5 (20)	81.2 (112)
Feeling worried/sad/unhappy, %	30.3 (40)	76.5 (101)	28.3 (39)	77.5 (107)
*Proxy*		*n* = 141		*n* = 147
Mobility, %	41.8 (59)	91.5 (129)	40.1 (59)	93.2 (137)
Looking after myself, %	51.8 (73)	90.1 (127)	50.3 (74)	89.8 (132)
Usual activity, %	27.7 (39)	87.2 (123)	27.2 (40)	87.8 (129)
Pain/discomfort, %	16.3 (23)	75.9 (107)	15.0 (22)	75.5 (111)
Feeling worried/sad/unhappy, %	23.4 (33)	68.1 (96)	21.8 (32)	67.3 (99)

OHA – overall health assessment

## Data Availability

The data that support the findings of this study are openly available in ‘Mendeley’ at: Wenjing, Zhou (2024), “Psychometric Validation of the Chinese Versions of EQ-5D-Y-3L and EQ-TIPS In Children and Adolescents with COVID-19”, Mendeley Data, V1, doi: 10.17632/w4xxkf7brg.1.

## Code availability

The computer code of this study are available from the corresponding author upon reasonable request.
